# CKAP2 (cytoskeleton-associated protein2) is a new prognostic marker in HER2-negative luminal type breast cancer

**DOI:** 10.1371/journal.pone.0182107

**Published:** 2017-08-03

**Authors:** Sung Hoon Sim, Chang-Dae Bae, Youngmi Kwon, Hai-Li Hwang, Shiv Poojan, Hye-In Hong, Kyungtae Kim, Seo-Hee Kang, Han-Seong Kim, Tae-Hyun Um, In Hae Park, Keun Seok Lee, So-Youn Jung, Seeyoun Lee, Han-Sung Kang, Eun Sook Lee, Mi-Kyung Kim, Kyeong-Man Hong, Jungsil Ro

**Affiliations:** 1 Center for Breast Cancer, National Cancer Center, 111 Jungbalsan-ro, Ilsandong-gu, Goyang-si, Gyeonggi-do, Republic of Korea; 2 Research Institute, National Cancer Center, 111 Jungbalsan-ro, Ilsandong-gu, Goyang-si, Gyeonggi-do, Republic of Korea; 3 Department of Molecular Cell Biology, Sungkyunkwan University School of Medicine, Suwon, Gyeonggi-do, Republic of Korea; 4 Department of Pathology, Inje University Ilsan Paik Hospital, Ilsanseo-gu, Goyang-si, Gyeonggi-do, Republic of Korea; 5 Department of Laboratory Medicine, Inje University Ilsan Paik Hospital, Ilsanseo-gu, Goyang-si, Gyeonggi-do, Republic of Korea; University of South Alabama Mitchell Cancer Institute, UNITED STATES

## Abstract

**Background:**

Recently, we reported cytoskeleton-associated protein2 (CKAP2) as a possible new prognostic breast cancer marker. However, it has not yet been applied in clinic. Therefore, clinical significance of CKAP2 was evaluated in comparison with that of Ki-67 in a cohort of breast cancer patients, and the expression difference was analyzed in cell cycle-arrested cancer and fibroblast cells.

**Methods:**

A total of 579 early breast cancer patients who underwent surgery at the National Cancer Center Hospital in Korea between 2001 and 2005 were accrued. CKAP2-positive cell count (CPCC) and Ki-67 labeling index (Ki-67LI) were evaluated by immunohistochemcal staining. The immunocytochemical staining patterns of CKAP2 and Ki-67 were analyzed in HeLa and human fibroblast cells after synchronization by double thymidine block.

**Results:**

Although there was a significant correlation (R = 0.754, *P* < 0.001) between CPCC and Ki-67LI, only CPCC was correlated with DFS in overall population (HR, 2.029; 95% CI, 1.012–4.068; *P* = 0.046) and HER2-negative luminal subgroup (HR, 3.984; 95% CI, 1.350–11.762; *P* = 0.012) by multivariate analysis. In immunocytochemical staining, more than 50% of serum-starved or non-mitotic cell phase HeLa cells were positive for Ki-67, in comparison to the low CKAP2-positivity, which might explain the prognostic difference between CPCC and Ki-67LI.

**Conclusions:**

The current study showed that CPCC but not Ki-67LI is an independent prognostic indicator in early breast cancer, more specifically in HER2-negative luminal breast cancer. The difference between two markers may be related to the lower background expression of CKAP2 in cancer cells.

## Introduction

Proliferation activity of cancer cells has long been suggested as a prognostic indicator in breast cancer [1], that genes related to the cell proliferation were incorporated in microarray gene sets for the prognostic prediction [2, 3]. Proliferation activity has also been used as a predictive marker to determine treatment options in early breast cancer [4, 5]. Mitotic index, which is measured by direct counting of mitotic cells, has been one of the most reliable prognostic markers in breast cancer [6, 7]. However, it has not been widely applied in the clinic, and useful immunohistochemical markers for the proliferation activity are in need.

Various proliferation markers such as cyclin D, p21 and Ki-67 have been tried to replace mitotic index [8]. Among them, Ki-67 has been most often studied, and its labeling index (Ki-67LI) has been suggested as a prognostic indicator in many studies [9, 10]. However, Ki-67 LI has not been used in routine clinical practice, because some studies failed to show its prognostic significance in the absence of standardization [11, 12]. Therefore, useful proliferation markers of prognostic significance are still in need.

Cytoskeleton-associated protein 2 (CKAP2) is a microtubule-associated protein which plays key roles in microtubule assembly and disassembly [13]. The expression level increases during cell proliferation, and is the highest in a mitotic phase [14]. In addition, the presence of CKAP2 protein appears in various locations depending on cell cycle phases: it starts to appear in the cytoplasm during G2 phase, and moves into mitotic spindle. Then, it is moved to condensed chromatin during mitotic phase, and disappears after the cytokinesis [15]. Nuclear CKAP2 expression appears most during mitotic phase, and chromatin CKAP2-positive cell count (CPCC) was well correlated with mitotic count suggesting it as a marker for proliferation activity [16]. In our previous study, CPCC was shown as a significant prognostic indicator in early breast cancer patients [17]. However, more data are needed to verify the significance of CPCC in correlation with survival within a consecutive tumor series under unified new tumor staging system. In addition, we assessed it in comparison with Ki-67LI. To analyze the discrepancy showing a difference in prognostic significance between two markers, CPCC and Ki-67LI, the expression patterns of both CKAP2 and Ki-67 were analyzed in cell cycle-arrested HeLa and human fibroblast cells.

## Materials and methods

### Patients and materials

Early breast cancer patients who underwent definitive surgery at the National Cancer Center Hospital between January 2001 and December 2005 were accrued. Patients who received neoadjuvant chemotherapy or without enough tumor tissues for the study were excluded. The study protocol was approved by the Institutional Review Board of National Cancer Center, Korea (NCCNCS-12-630), and IRB waived the need for consent.

### Immunohistochemistry and evaluation of CPCC and Ki-67LI

Immunohistochemical staining on formalin-fixed, paraffin-embedded tumor tissues was performed using Ki-67 antibodies (Ki-67 IgG; Thermo Scientific, MA, USA) and CKAP2 antibody as previously described[16]. Briefly, after deparaffinization of formalin-fixed, paraffin-embedded tissues, antigen was retrieved in 10 mM citrate buffer containing 0.05% Tween 20. After ethanol fixation, the tissues were treated with 3% hydrogen peroxide and Ultra V block solution. After 1 h room-temperature incubation with anti-CKAP2 antibody, the slides were washed in Tris-buffered saline including Tween 20 (TBST), incubated with primary antibody enhancer for 10 min, and exposed to horseradish peroxidase-conjugated secondary antibody for 15 min. After re-washing in TBST, the tissue slides were incubated with diaminobenzidine chromogen (Scytek Laboratories Inc, Logan, UT) and counter-stained with Mayer’s hematoxylin (Dako Cytomation, Glostrup, Denmark).

The CPCC were calculated by summing the numbers of chromatin CKAP2-positive cells identified under 10 consecutive high-power fields (x400, 1 HPF = 0.16 mm2) in the areas showing the highest number of chromatin CKAP2-positive cells, as reported in our previous study [16]. Ki-67LI was determined as percentage after counting the total number of positive cells per 1000 tumor cells.

### Cell cycle synchronization using double thymidine block and preparation of thromboplastin-plasma cell-block

Cultured HeLa cells (CCL-2, ATCC, Manassas, VA) or human fibroblast cells (# SCC058, Millipore, Darmstadt, Germany) at 30% confluency were washed twice with phosphate buffered saline, and thymidine double block was performed as previously reported [16]. After two cycles of thymidine treatment, the cells were incubated in culture media. At 0, 2, 4, 6, 7, 8, 10 h, the cells were harvested, and fixed in 95% ethanol. The cells incubated in serum-free media for 24 h, 48 h, or 72 h were used as non-proliferating controls. Thromboplastin-plasma cell-blocks were prepared for the immunohistochemical analysis, as previously reported [16]. The cells were also employed for flow cytometry to check the synchrony after propidium iodide staining.

### Western blot analysis

Cells were lysed in lysis buffer (20 mM Tris, pH 7.4, 5 mM EDTA, 10 mM Na_4_P_2_O_7_, 100 mM NaF, 1% NP-40, 1 mM PMSF, 0.2% protease inhibitor cocktail and phosphatase inhibitor). The protein concentrations of the cell lysates were measured using the Pierce BCA Protein Assay kit (Pierce, Rockford, USA). Protein lysates were then resuspended in loading buffer, boiled for 5 minutes, subjected to SDS-PAGE, and immunoblotted with antibodies. In addition to CKAP2 antibody, antibodies for phospho-S10-histone H3 (ab14955, Abcam, Cambridge, UK, 1:500), Rb (#9309, Cell Signaling, Danver, MS, 1:2000), phospho-Rb-S807, S811 (#9308, Cell Signaling, 1:1000), cyclin D1 (sc-246, Santa Cruz, Dallas, TX, 1:1000), cyclin E (sc-481, Santa Cruz, 1:1000), cyclin A (sc-751, Santa Cruz, 1:1000), cyclin B1 (#4138, Cell Signaling, 1:1000), and GAPDH (ab8245, Cell Signaling, 1:2500) were used.

### Statistical analysis

The association between CPCC and Ki-67LI was analyzed by Spearman’s rank correlation test. The cutoff values for CPCC and Ki-67LI were determined by using time-dependent receiver operating curve (ROC) analysis (R software version 3.2.3, The R foundation for statistical computing, Vienna, Austria. http://www.r-project.org). The CPCC and Ki-67LI were classified as high (CPCC>7; Ki-67LI >8) or low (CPCC≤7; Ki-67LI ≤8). Disease free survival (DFS) was defined as the time from the radical surgical resection to the diagnosis of relapse, or the last date of follow-up. The Kaplan-Meier method was used for the estimation of DFS, and the difference in DFS was analyzed by log-rank test. Multivariate analysis was performed using Cox proportional hazards modeling to determine hazard ratios with their 95% confidence intervals (CI). A two sided value of *P* < 0.05 was considered as statistically significant.

## Results

### Clinical characteristics of accrued patients

From the hospital database between 2001 and 2005, 681 consecutive breast cancer patients who underwent surgery without receiving neoadjuvant treatment were identified. One hundred two cases were excluded due to the following reasons: angiosarcoma (n = 1), distant metastasis (n = 3), ductal carcinoma *in situ* (n = 5), and insufficient tumor tissues for immunohistochemistry (n = 93). Clinical characteristics of final 579 patients are shown in Table 1. The majority of patients had invasive ductal carcinoma (88.9%) and stage I/II disease (92.9%) with a median age of 45 years (range 26–82). Breast cancer subtypes by immunohistochemical method were identified in 340 cases: HER2 negative luminal type (HER2-negative and hormone receptor positive), HER2 positive type (HER2 positive irrespective of hormone receptors status), and triple negative (TN) type (negative for hormone receptors and HER2) were 205 (35.4%), 103 (17.8%), and 32 (5.5%), respectively. Median follow-up time was 116.8 months and recurrence was observed in 65 patients. Mean DFS was 152.2 months (95% CI, 148.0–155.6).

**Table 1:** Clinicopathologic characteristics.

CLINICAL VARIABLES	TOTAL N = 579	N(%)
Age	median (range)	45 (26~82)
Sex	Male	0 (0)
	Female	579 (100)
Histology	Invasive carcinoma	
	ductal	515 (88.9)
	lobular	23 (4.0)
	Others	41 (7.1)
Histologic grade	Grade 1	72 (12.4)
	Grade 2	266 (46.0)
	Grade 3	198 (34.2)
	Unknown	43 (7.4)
T stage	1	416 (71.8)
	2	159 (27.5)
	3	3 (0.5)
	4	1 (0.2)
N stage	0	376 (64.9)
	1	164 (28.3)
	2	27 (4.7)
	3	12 (2.1)
ER/PR	Positive	472 (81.5)
	Negative	106 (18.3)
	Unknown	1 (0.2)
HER2/*neu*	Positive	103 (17.8)
	Negative	237 (40.9)
	Unknown	239 (41.3)
Subgroup	luminal type (HER2 negative)^a^	205 (35.4)
	HER2 positive^b^	103 (17.8)
	Triple negative^c^	32 (5.5)
	Unknown	239 (41.3)
Adjuvant therapy	Chemotherapy	422 (72.9)
	Hormone	462 (80.3)
	Trastuzumab	10 (1.7)
CPCC (median, range)		8 (0–170)
Ki-67LI(%) (median, range)		10.2(0–91.7)

aHER2-negative luminal case: hormone receptor (HR)-positive and HER2-negative

bHER2-positive case: HER2-positive status irrespective of HR status

cTriple-negative (TN) case: HR-negative and HER2-negative

ER = estrogen receptor; PR = progesterone receptor; HR = hormone receptor; HER2 = human epidermal growth factor receptor 2; CPCC = chromatin CKAP2-positive cell count; Ki-67LI = Ki-67 labeling index

### Immunohistochemical analysis of CPCC and Ki-67LI and its relationship with clinical characteristics

**Fig 1:**
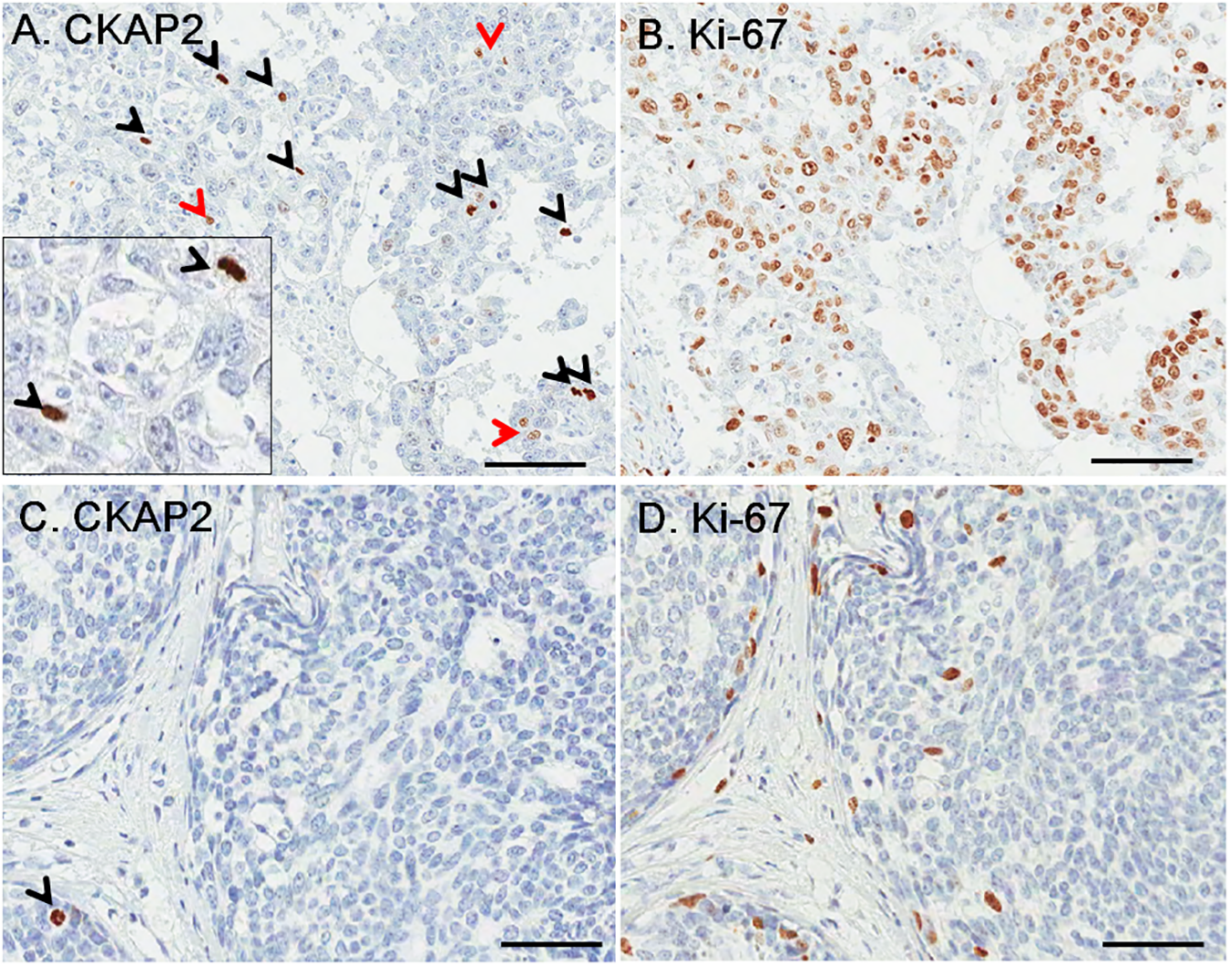
Immunohistochemical staining for CKAP2 and Ki-67 in breast cancer tissues. A case with higher CKAP2-positive cells (A) showed higher Ki-67-positive cells (B). A case with lower CKAP2-positive cells (C) showed lower Ki-67-positive cells (D). The black arrow heads indicate cells with positive chromatin CKAP2 staining. The red ones indicate post-cytokinetic cells. Higher magnification for CKAP2 staining was shown in the box for A. One hundred μm rulers are shown.

Chromatin CKAP2 staining was evident in mitotic cells, and the number of chromatin CKAP2-positive cells were variable among breast cancer cases (Fig 1A and 1C). Higher number of Ki-67 positive nuclei than CKAP2 was observed (Fig 1B and 1D), indicating that Ki-67 is expressed in multiple cell phases during proliferation period. CPCC (median, 8; range, 0–170) and Ki-67LI (median 10.2; range, 0–91.7%) were significantly correlated (R = 0.754, P < 0.001).

**Fig 2:**
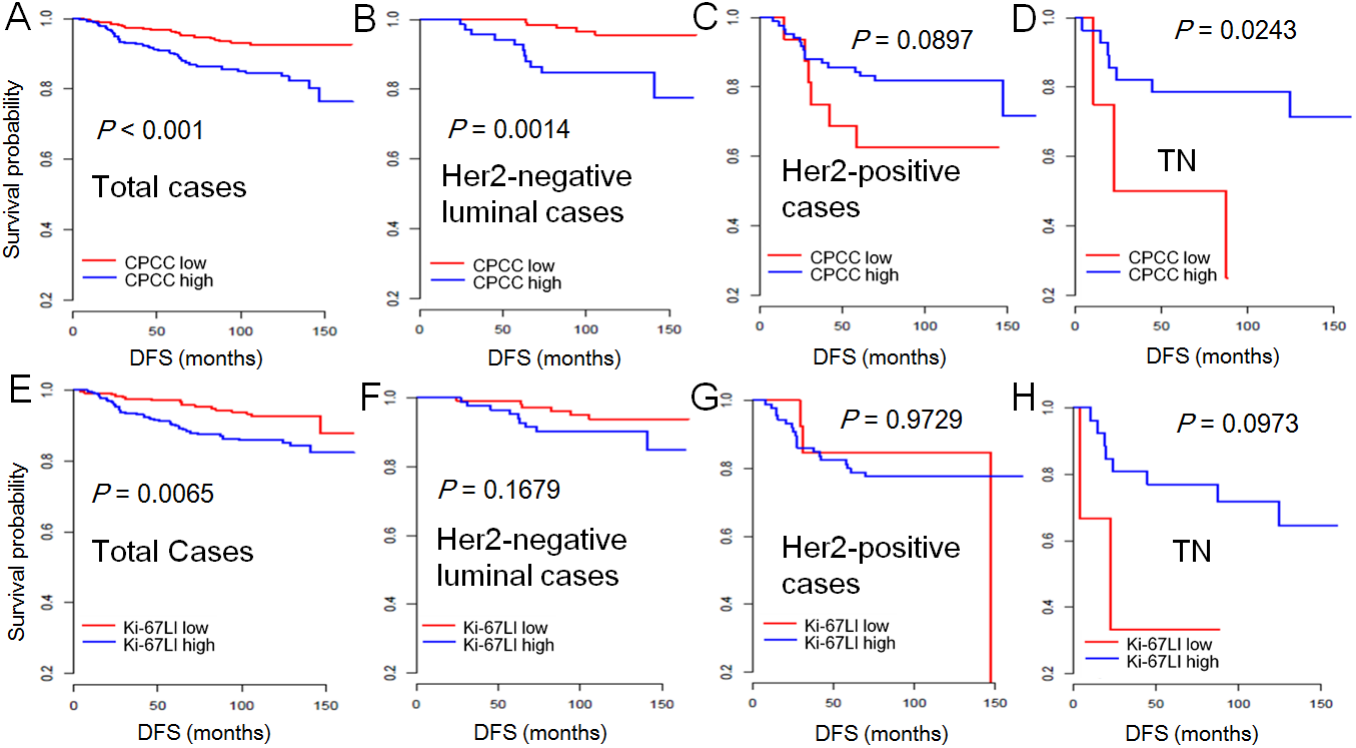
Kaplan-Meier plot for CPCC and KI-67LI in breast cancer. CPCC showed significant correlations with poor DFS in total cases (A) or HER2-negative luminal cases (B), but neither in HER2-positive (C), nor in triple-negative cases (D). Ki-67LI showed a significant correlation with poor DFS in total cases (E), but not in any subgroups such as HER2-negative luminal cases (F), HER2-positive (G), and triple-negative cases (H). The *P* value was determined by log-rank test. The X-axis is DFS in months, and the Y-axis, DFS probability. CPCC = chromatin CKAP2-positive cell count; Ki-67LI = Ki-67 labeling index.

In Kaplan-Meier plot, high CPCC and Ki-67LI both showed significant correlations with poor DFS (Fig 2A and 2E). In the univariate analysis of total cases, both were also significantly correlated with poor survival (*P* = 0.001 for CPCC, *P* = 0.008 for Ki-67LI, Table 2). Of all clinicopathologic factors, T stage, N stage, estrogen receptor (ER) and progesterone receptor (PR) status were significantly associated with DFS (Table 2). In multivariate analysis considering co-variables including age, T stage, N stage, ER and PR status, high CPCC was significantly associated with poor DFS (HR = 2.029, 95% CI = 1.012–4.068, *P* = 0.046), whereas Ki-67LI was not (HR = 1.602, 95%CI = 0.787–3.263, *P* = 0.194, Table 3).

**Table 2:** Univariate analysis of clinicopathological factors in 579 early breast cancer cases.

Clinical Variables	Subgroups	Total Patients				HER2-negative luminal subgroup			
		N	HR	95%CI	*P*	N	HR	95%CI	*P*
Age		579	0.978	0.950–1.006	0.116	205	0.948	0.888–1.013	0.116
T	1	416	1			152	1		
	≥2	163	2.306	1.415–3.756	<0.001	53	3.124	1.171–8.334	0.023
N	0	376	1			126	1		
	≥1	203	2.071	1.272–3.369	0.003	79	2.123	0.790–5.700	0.135
Histologic	IDC	515	1			182	1		
	ILC	23	1.571	0.570–4.328	0.382	13	0.988	0.130–7.520	0.991
	Others	41	0.677	0.212–2.162	0.511	10	0.000	0-∞	0.991
Histologic Grade	Grade 1	72	1			42	1		
	Grade 2	266	1.781	0.614–5.171	0.288	110	4.742	0.603–37.306	0.139
	Grade 3	198	3.222	1.131–9.180	0.029	40	5.141	0.573–46.107	0.144
ER	Negative	115	1						
	Positive	463	0.417	0.250–0.693	<0.001				
PR	Negative	168	1						
	Positive	410	0.332	0.204–0.541	<0.001				
HER2/neu	Negative	237	1						
	Positive	103	2.607	1.559–4.359	<0.001				
Adjuvant Chemotherapy	Yes	422	1			158	1		0.389
	No	157	0.593	0.310–1.134	0.114	47	0.522	0.118–2.296	
Adjuvant hormone therapy	Yes	465	1			199	1		<0.001
	No	114	2.574	1.547–4.281	<0.001	6	8.497	2.419–29.845	
Adjuvant anti-HER2 therapy	Yes	10	1						
	No	569	0.529	0.129–2.161	0.375				
Subgroup	HER2 Negative luminal^*^	205	1						
	HER2 positive^**^	103	3.203	1.867–5.494	<0.001				
	Triple Negative^***^	32	4.927	2.426–10.006	<0.001				
	Unknown	239							
Ki-67LI	≤8	244	1			116	1		
	>8	325	2.127	1.219–3.711	0.008	87	2.038	0.725–5.725	0.177
CPCC	≤7	281	1			134	1		
	>7	296	2.517	1.474–4.296	<0.001	71	4.789	1.662–13.799	0.004

*HER2-negative luminal subgroup: cases with hormone receptor (HR)-positive and HER2-negative status

**HER2-positive subgroup: HER2-positive status and HR negative

***Triple-negative subgroup (TN): HR-negative and HER2-negative status

CI = confidence interval; ER = estrogen receptor; PR = progesterone receptor; HR = hormone receptor; HER2 = human epidermal growth factor receptor 2; CPCC = chromatin CKAP2-positive cell count; Ki-67LI = Ki-67 labeling index

**Table 3:** Multivariate analysis of CPCC or Ki-67LI for correlation with DFS in breast cancer subgroups by Cox proportional hazard regression model.

Parameter	All Patients^1^)		HER2-negative luminal^2^)		HER2-positive (HR negative) ^3^)		Triple negative^4^)	
	HR (95% CI)	*P*	HR (95% CI)	*P*	HR (95% CI)	*P*	HR (95% CI)	*P*
CPCC (≤7 vs >7)	2.029 (1.012–4.068)	0.046	3.984 (1.350–11.762)	0.012	0.432 (0.166–1.126)	0.086	0.214 (0.051–0.893)	0.034
Ki-67LI (≤8 vs >8)	1.602 (0.787–3.263)	0.194	1.788 (0.595–5.370)	0.301	1.052 (0.309–3.586)	0.935	0.265 (0.043–1.631)	0.152

For the analysis, the co-variables of age at the diagnosis, T stage (T1, T≥2), N stage (N0, N≥1), hormone receptor status (ER/PR status), histologic grade, and adjuvant chemotherapy for all patients^1^), the co-variables of age at the diagnosis, T stage (T1, T≥2), N stage (N0, N≥1), adjuvant chemotherapy, and adjuvant hormone therapy for HER2-negative luminal cases^2^), and the covariables of age at the diagnosis, T stage (T1, T≥2), N stage (N0, N≥1), and adjuvant chemotherapy for HER2-positive^3^), or triple negative^4^) subgroups were used in the multivariate analysis, treating each co-variable as a categorical variable, except for the variable of age at the diagnosis which is treated as a continuous variable.

In the subgroup univariate analyses, CPCC (*P* = 0.004), T stage (*P* = 0.023), and adjuvant hormone therapy (*P* = 0.001) showed significant associations with DFS in HER2-negative luminal subtype (Table 2, Fig 2B). However, Ki-67LI did not show any significant correlation with DFS (*P* = 0.177, Fig 2F). In the multivariate analysis of HER2-negative luminal subtype considering variables such as age, T stage, N stage and adjuvant treatment, high CPCC was significantly associated with poor DFS (HR, 4.789; 95%CI, 1.662–13.799; *P* = 0.004), whereas the Ki-67LI was not (Table 3), suggesting that CPCC is a significant survival indicator, and CKAP2 is a better prognostic marker than Ki-67 in HER2-negative luminal subtype of breast cancer. In other subgroup analyses, CPCC showed significance also in triple negative patients (*P* = 0.034, Table 3). Ki-67LI showed no significance in any subtypes (Table 3).

### Expression of CKAP2 and Ki-67 in various cell cycle phases

**Fig 3:**
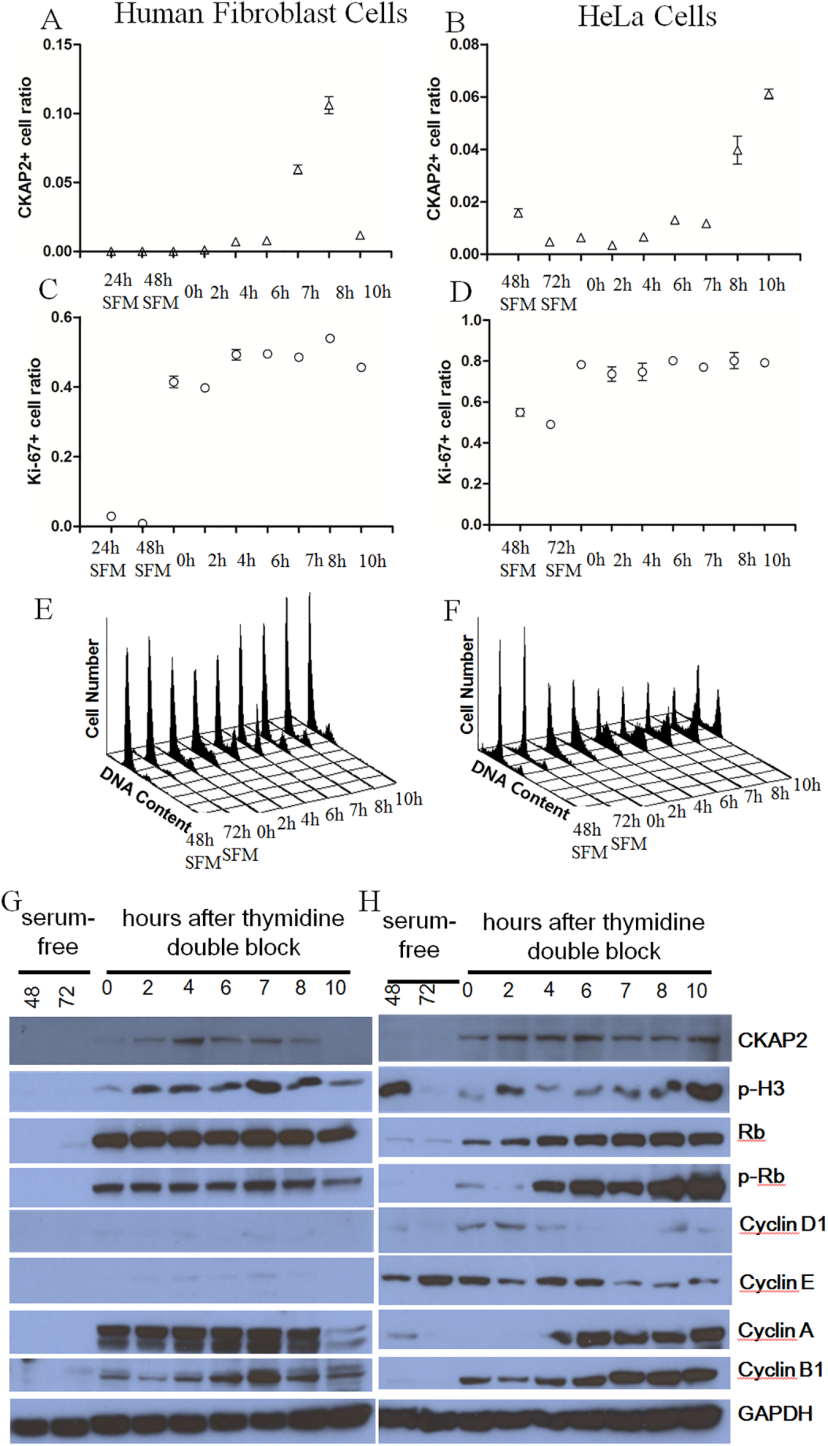
Ki-67-positive or CKAP2-positive cell ratios in human fibroblast and HeLa cells after cell cycle synchronization. Peak chromatin CKAP2 positive cell rates were observed in 7–8 hours after thymidine double block for HeLa cell (A), and in 8–10 hours for human fibroblast cell (B). In contrast, peak Ki-67-positive cell rate was not clear in human fibroblast (C) and HeLa cells (C). In serum starved HeLa cells for 48 (48 SFM), 72 (72 SFM or serum free media) hours, Ki-67-positive cell rate was about 50%, in contrast to low CKAP2-positive rate. The CKAP2 or Ki-67 positive ratios were determined by calculating the positive cells out of total cells, and counting positive cells were performed three times. Cell cycle synchrony was shown in human fibroblast cells (E) and HeLa cells (F). Expression patterns of CKAP2 and cell cycle dependent proteins such as phospho-S10-histone 3 (p-H3), Rb, phospho-Rb-S807, S811 (p-Rb), cyclin D1, cyclin E, cyclin A, cyclin B1, and GAPDH in various cell phases in human fibroblast (G) and HeLa cell (H) was analyzed by Western blot. Cell cycle dependent proteins for Western blot analysis were indicated on the right side of each strip. x-axis in A-D, the release time after the double thymidine block or incubation time in serum free media for 24, 48, or 72 h; y-axis in A-D, chromatin CKAP2-positive or Ki-67-positive cell ratios. For E and F, the number of cells was plotted against DNA content at the indicated release time points after the double thymidine block. Serum-starved samples for 48 h (48) and 72 h (72), and samples cultured for indicated hours (0, 2, 4, 6, 7, 8, and 10) after thymidine double block were indicated at the top of G and H.

In the analysis of human fibroblasts after the cell synchronization by thymidine double block, CKAP2 staining was observed mostly in mitotic cell phase (Fig 3A). However, Ki-67 was positively stained in 40–50% of human fibroblast cells consistently in various cell cycle phases (Fig 3C).

In the analysis of HeLa cells after cell synchronization, CKAP2 was again majorly mitotic phase-specific than Ki-67 (Fig 3B and 3D). In addition, Ki-67 positive cell rate of HeLa cell was around 50% in serum-free media condition (Fig 3D), whereas CKAP2-positive rate was quite low in the same condition (Fig 3B). In serum-free media condition of human fibroblast cell, Ki-67 positive cell rate was very low (Fig 3C). The FACS data (Fig 3E and 3F) and immunocytochemical staining results (S1 Fig) are shown for both CKAP2 and Ki-67 in various cell phases.

On Western blot analysis, expression of phopho-H3 or phospho-Rb which is specific to M phase cells was peak at 10 h for HeLa and 7 h for human fibroblast after thymidine double block (Fig 3G and 3F), and the peak times of phopho-H3 expression was quite consistent with those at peak chromatin CKAP2-positive cell ratios in immunocytochemistry (Fig 3A and 3B).

On Western blot analysis of serumstarved human fibroblast cells, no expression of CKAP2 and Ki-67 was observed. In HeLa cells after 48 hour serum starvation, however, phospho-H3 was clearly observed in contrast to no expression of CKAP2, suggesting that phosphorylation in H3 has not been removed during the first 48 hours after the serum starvation, which is quite similar to Ki-67 in immuno-cytochemical staining (Fig 3D) where Ki-67 expression persisted for 48–72 hours after serum starvation. These results indicate that both phospho-H3 and Ki-67 in some cancer cells would persist aberrantly after cell division.

## Discussion

In the current study, it was shown that high CPCC was a significant independent prognostic marker for recurrence in early breast cancer patients, and appeared to be a superior prognostic marker to Ki-67LI. In the subgroup analyses, CPCC was significantly associated with recurrence in HER2-negative luminal subtype, but not in HER2-positive subtype.

The current study result of a significant association of CPCC with survival is consistent with our previous report [17]. This significance of CPCC may be based on its high correlation with MAI as shown in a previous study [16]. Ki-67LI was highly correlated with MAI, but was not an independent prognostic factor. Previously others reported that MAI was a reliable prognostic marker for breast cancer, while Ki-67LI was not [6, 7, 12, 18], which are in line with our data.

To clarify the reason for the difference in prognostic significance between CKAP2 and Ki-67, immunocytochemical analysis was performed using cells in various cell phases in the present study. In the analysis, we found that chromatin CKAP2 staining was fairly restricted to the cells in the mitotic phase as previously reported [17]. In contrast, nuclear Ki-67 staining was observed across all phases from G1 to post-mitotic phases of proliferating cells, which may be a basis of explanation for the difference in predicting prognosis. In addition, we found that, in non-proliferating or serum-starved HeLa cells, baseline Ki-67LI was high, around 50%, but CPCC was quite low, suggesting the persistence of Ki-67 at non-proliferating status after the completion of cell cycle. On Western blots of HeLa cells, phospho-H3 persisted after 48 hours of serum-starvation, which is quite similar to Ki-67 persistence. Cancer cells tend to have aberrant cell cycle regulation, and the perturbation of cell cycle may delay the removal of phospho-H3 or Ki-67 as shown in the present study. In contrast, the removal of CKAP2 does not seem to be significantly affected even in cancer cells. Therefore, our result suggests that the removal of proliferation markers can be delayed due to the aberrant cell cycle regulation in cancer cells, and can be a significant factor in the evaluation of proliferation activity.

Although the clinical significance of proliferation activity in the subgroups of breast cancer patients has not been well defined [17], CPCC showed a significant correlation with survival, especially in HER2-negative luminal subtype of patients but not in HER2-positive subtype. This suggests that the proliferation activity may have more clinical impact in the HER2-negative luminal subtype of breast cancer patients. Our result is in line with the St. Gallen Consensus recommendation on the use of a proliferation marker, Ki-67, in order to classify the hormone receptor-positive luminal subtype [2]. It also suggests that chemotherapy could be considered in patients with high CPCC due to their worse survival. Although CPCC showed a significant correlation with DFS in the TN subgroup in the current study (Fig 2), the sample size is insufficient and further study in triple negative breast cancer is warranted.

This study has several limitations. First, this is a retrospective cohort study, and further prospective cohort studies are needed for the validation. Second, the majority of HER2-positive patients in the present study did not receive anti-HER2 adjuvant treatment. Third, the present CPCC could have inter-observational variability as Ki-67LI does. Of note, it was reported that the variability of CPCC was far less than MAI [16, 17].

## Conclusion

In conclusion, the current study showed that CPCC is a significant prognostic indicator for disease free survival in HER2-negative luminal subtype of breast cancer patients. Since CKAP2 appears to be more specific to actively proliferating cells, CPCC could be an alternative option or a complementary to Ki-67LI or MAI as a prognostic marker in breast cancer.

## Supporting information

S1 Fig Immunohistochemical staining in synchronized cells or cells incubated in serum free media (SFM). Human fibroblast cells (A-F) or HeLa cells (G-L) were synchronized by thymidine double block, and released from the block at 0 h (B, E, H, and K), 8 h (C and F), or 10 h (I and L). CKAP2 (A-C, and G- I) or Ki-67 immunostaining (D-E, and J-L) was performed. The arrow heads indicate cells with positive chromatin CKAP2 staining. One hundred μm rulers are shown.
(DOCX)
