# Supplementary material for: CKAP2 (cytoskeleton-associated protein2) is a new prognostic marker in HER2-negative luminal type breast cancer
Source: PLoS One. 2017 Aug 3;12(8):e0182107. doi: 10.1371/journal.pone.0182107 (PMC5542386; doi:10.1371/journal.pone.0182107)
Supplement: S1 Fig — Human fibroblast cells (A-F) or HeLa cells (G-L) were synchronized by thymidine double block, and released from the block at 0 h (B, E, H, and K), 8 h (C and F), or 10 h (I and L). CKAP2 (A-C, and G- I) or Ki-67 immunostaining (D-E, and J-L) was performed. The arrow heads indicate cells with positive chromatin CKAP2 staining. One hundred μm rulers are shown. (DOCX) [file pone.0182107.s001.docx]

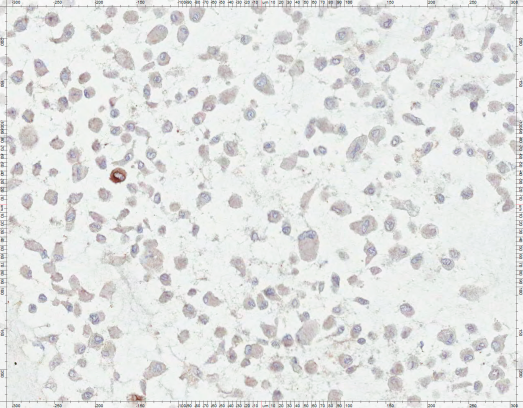

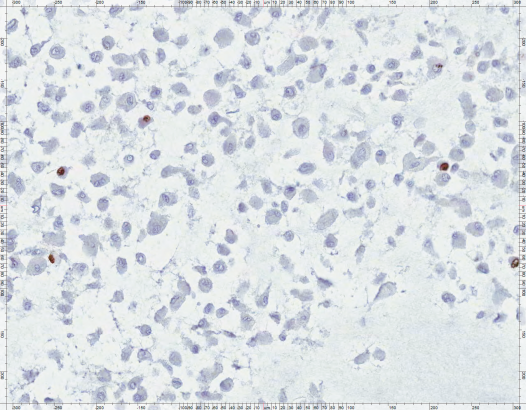

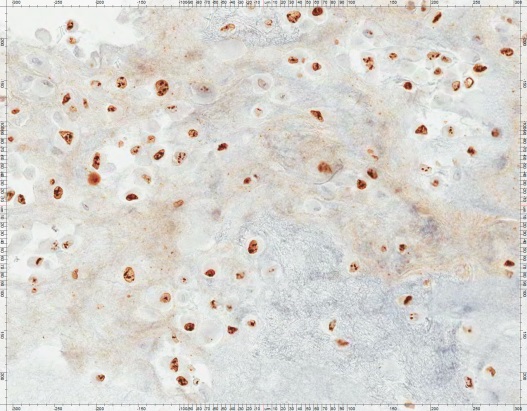

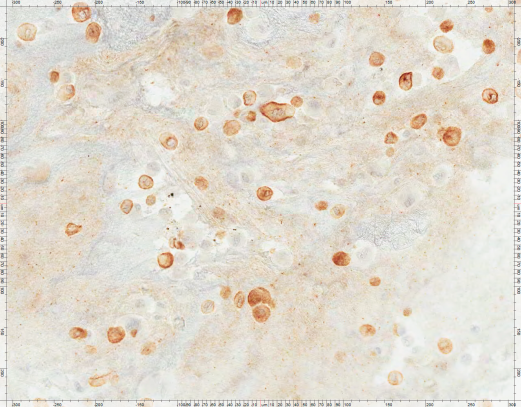

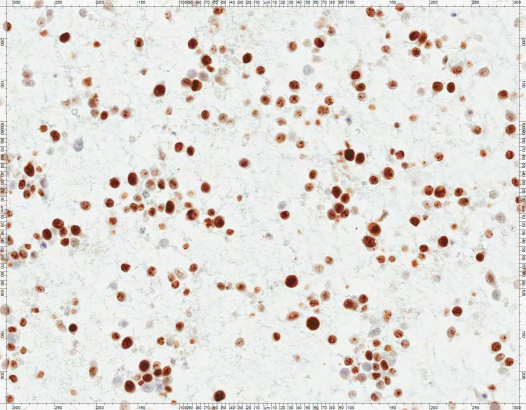

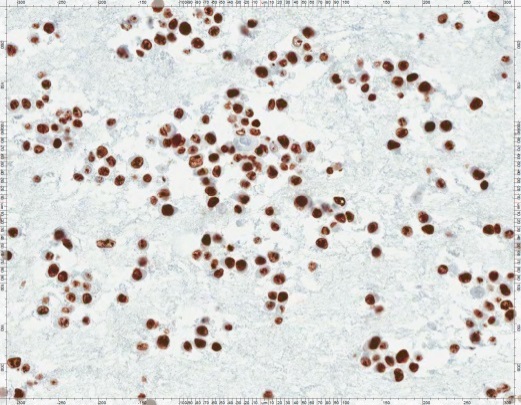

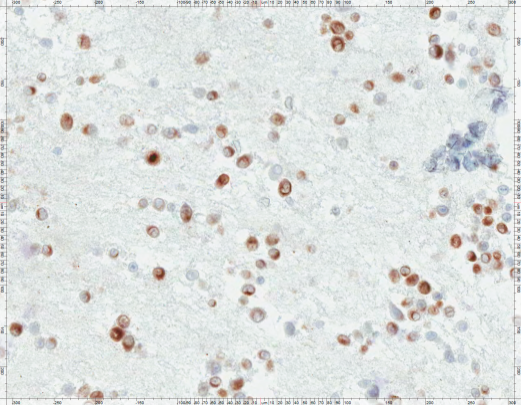

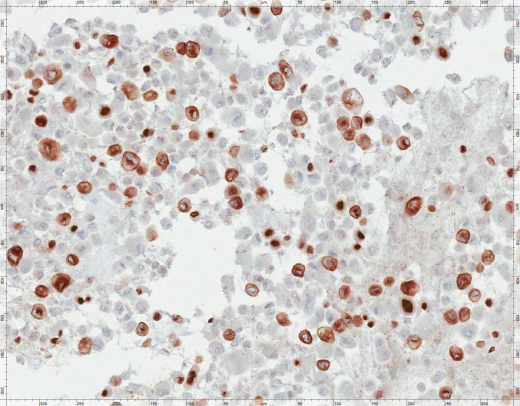

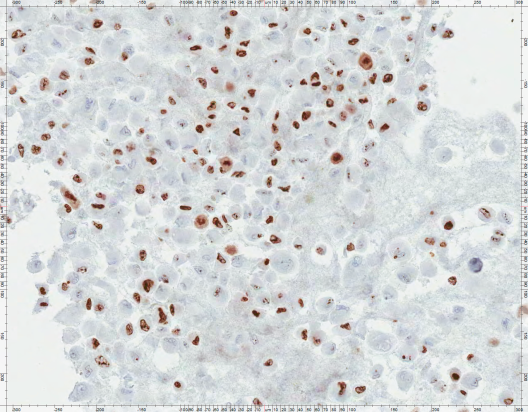

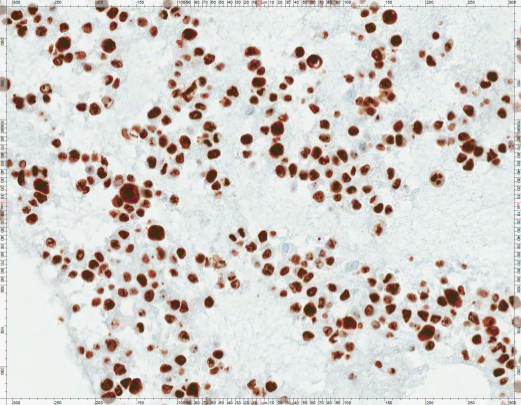

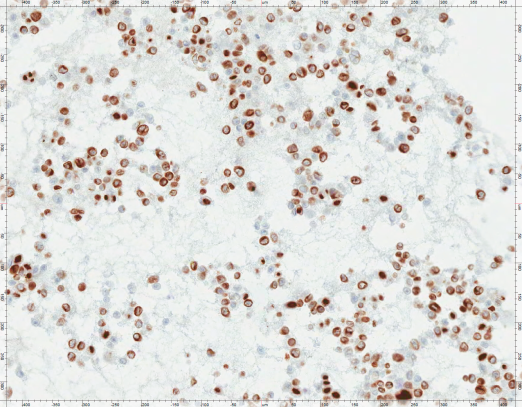

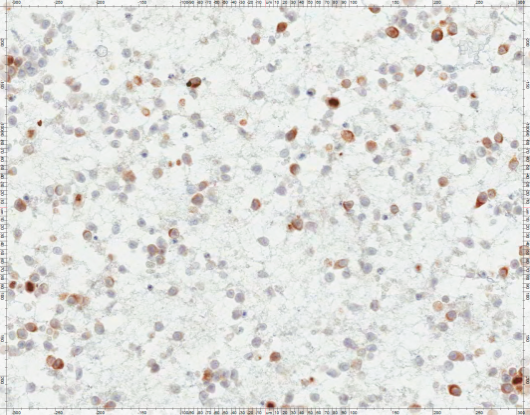


SFM

0h

8 or 10 h

**Human Fibroblast Cell**

**HeLa Cell**

A

B

C

D

E

F

G

H

I

J

K

L

G

**CKAP2**

**Ki-67**

**CKAP2**

**Ki-67**

Figure S1. Immunohistochemical staining in synchronized cells or cells incubated in serum free media (SFM). Human fibroblast cells (A-F) or HeLa cells (G-L) were synchronized by thymidine double block, and released from the block at 0 h (B, E, H, and K), 8 h (C and F), or 10 h (I and L). CKAP2 (A-C, and G- I) or Ki-67 immunostaining (D-E, and J-L) was performed. The arrow heads indicate cells with positive chromatin CKAP2 staining. One hundred μm rulers are shown.
